# Cross-Linguistic Influence in the Bilingual Mental Lexicon: Evidence of Cognate Effects in the Phonetic Production and Processing of a Vowel Contrast

**DOI:** 10.3389/fpsyg.2016.00617

**Published:** 2016-04-26

**Authors:** Mark Amengual

**Affiliations:** Bilingualism Research Laboratory, Department of Languages and Applied Linguistics, University of CaliforniaSanta Cruz, CA, USA

**Keywords:** bilingualism, speech production, speech processing, cross-linguistic influence, mental lexicon, cognates, lexical storage

## Abstract

The present study examines cognate effects in the phonetic production and processing of the Catalan back mid-vowel contrast (/o/-/ɔ/) by 24 early and highly proficient Spanish-Catalan bilinguals in Majorca (Spain). Participants completed a picture-naming task and a forced-choice lexical decision task in which they were presented with either words (e.g., /bɔsk/ “forest”) or non-words based on real words, but with the alternate mid-vowel pair in stressed position (^*^/bosk/). The same cognate and non-cognate lexical items were included in the production and lexical decision experiments. The results indicate that even though these early bilinguals maintained the back mid-vowel contrast in their productions, they had great difficulties identifying non-words and real words based on the identity of the Catalan mid-vowel. The analyses revealed language dominance and cognate effects: Spanish-dominants exhibited higher error rates than Catalan-dominants, and production and lexical decision accuracy were also affected by cognate status. The present study contributes to the discussion of the organization of early bilinguals' dominant and non-dominant sound systems, and proposes that exemplar theoretic approaches can be extended to include bilingual lexical connections that account for the interactions between the phonetic and lexical levels of early bilingual individuals.

## Introduction

A bilingual/multilingual individual must acquire two or more sound systems with differing sets of segments. Studies on the production and perception of language-specific phonological contrasts have examined early and late bilinguals differing in proficiency, age of acquisition, language dominance, amount of L2 input received, and other biographical non-linguistic variables in order to better understand cross-linguistic influence in bilingual speech (Flege, 1991, 2007; Flege et al., 1995, 1997, 1999; Guion, 2003; Flege and MacKay, 2004; Antoniou et al., 2011; Darcy and Krüger, 2012; Barlow, 2014; Simonet, 2014, 2015; Amengual and Chamorro, 2015, among others). In addition to producing and perceiving phonological categories specific to each of their languages, bilinguals need to be able to establish lexical representations in their dominant and non-dominant language that encode language-specific phonemic contrasts. Following this assumption, recent studies have explored the dimension of the phonology/lexicon interface as opposed to experimental paradigms that focus exclusively on the categorization of phones without necessarily testing their linguistic function. This line of research seeks to determine how bilingual speakers encode words in their mental lexicon, how bilinguals resolve an increase in lexical competition due to having phonological representations of words in two different languages, and the impact of non-robust phonological representations with regard to bilingual lexical access (Weber and Cutler, 2004; Cutler et al., 2006; Escudero et al., 2008; Hayes-Harb and Masuda, 2008; Darcy et al., 2012; Amengual, 2015).

Prior research also suggests that not all lexical items are accessed and retrieved the same way, providing evidence of lexical effects in language acquisition and use. Some of these well-documented lexical effects include word frequency effects (Oldfield and Wingfield, 1965; Dell, 1990; Brysbaert et al., 2011), lexical neighborhood density effects (Baese-Berk and Goldrick, 2009; Peramunage et al., 2011; Scarborough, 2012), lexical bias effects (Vigliocco and Harsuiker, 2002; Nooteboom, 2005; Oppenheim and Dell, 2008), and cognate status effects (Dijkstra et al., 1999; Lemhöfer et al., 2004). Cognates, generally defined as lexical items with considerable phonological, semantic, and orthographic similarity (de Groot, 1995, p. 167), represent “the lexical overlap between languages” (Lemhöfer et al., 2004, p. 587). Given that many language pairs have lexical items that share form and meaning, these cognate words are likely to have a special status for bilinguals.

Facilitation effects with cognates have been widely studied in bilingual populations, particularly in psycholinguistic research. Word recognition and word naming experiments have shown that L2 cognate words are translated more rapidly and accurately than non-cognates (de Groot, 1992a,b), that there is faster (and more accurate) lexical access for cognate words compared to non-cognates in lexical decision tasks (Caramazza and Brones, 1979; Dijkstra et al., 1998, 1999; de Groot et al., 2002), that cognates show greater repetition priming effects (Cristoffanini et al., 1986; Sánchez-Casas et al., 1992; de Bot et al., 1995), that cognates are easier to learn (de Groot et al., 2002), and that there are facilitatory effects of cognates in production (Costa et al., 2005), with cognates being named faster in word naming tasks (de Groot et al., 2002) and picture naming tasks (Costa et al., 2000; Hoshino and Kroll, 2008). Recent studies have also examined the effect of cognate status on the acoustic realization of phonetic segments, and the results support a cognate effect in bilingual speech production (Cochrane, 1980; Flege and Munro, 1994; Brown and Harper, 2009; Amengual, 2012; Mora and Nadeu, 2012; Goldrick et al., 2014; Brown and Amengual, 2015; Jacobs et al., 2016). These findings provide evidence of cross-language effects in the interface between the phonological and the lexical levels.

The phonetic variable under investigation in the present study is the Catalan-specific back mid-vowel contrast (/o/-/ɔ/), which exists in Catalan but not in Spanish. Catalan stressed vowels have four degrees of height; with salient differentiation in the mid-vowel area while the Spanish vowel system comprises the five cardinal vowels. There is a wealth of literature that has examined the production, perception, and processing of the Catalan mid-vowel contrasts showing that Spanish-Catalan bilinguals in Barcelona are merging /e/-/ε/ to /e/ and /o/-/ɔ/ to /o/ in their productions (i.e., producing Spanish-like mid-vowels) and they are reported to be failing to distinguish these Catalan-specific mid-vowel contrasts (Recasens, 1991; Pallier et al., 1997; Sebastián-Gallés and Soto-Faraco, 1999; Bosch et al., 2000). Furthermore, perception difficulties have been shown to also have consequences for lexical access. In a series of studies (Sebastián-Gallés and Baus, 2005; Sebastián-Gallés et al., 2005), Spanish-Catalan bilinguals in Barcelona participated in a lexical decision task involving Catalan words and non-words, in which non-words were based on real words but with the stressed vowel changed (i.e., the Catalan phoneme /e/ was substituted for /ε/, or vice versa). The results indicated that bilinguals in Barcelona had great difficulty distinguishing between words and non-words that differed by the Catalan front mid-vowel contrast (/e/-/ε/), and Spanish-dominants overall exhibited a higher error rate than Catalan-dominants. These earlier findings in Barcelona may have been an artifact of the variety of Catalan being acquired. The study of the Catalan mid-vowels of early bilinguals in a different bilingual community, such as the one in Majorca, provides the opportunity to considerably reduce confounding factors that could have affected the previous results with Spanish-Catalan bilinguals in Barcelona. Due to differences in the historical evolution of the vowel systems in the dialects of Catalan, Majorcan Catalan has a vowel system and lexical distribution of these vowels that is distinct from the variety spoken in Barcelona. In addition, the Catalan mid-vowel contrasts in Majorca are more robustly maintained in the productions of these bilinguals in comparison to those in Barcelona (Herrick, 2003, 2006, 2007, 2008; Carrera-Sabaté and Fernández-Planas, 2005; Recasens and Espinosa, 2006, 2009; Amengual, 2011, 2013, 2016; Simonet, 2011, 2014). In a bilingual setting such as the one in Barcelona, Spanish-dominant speakers may receive highly variable and inconsistent Catalan input (i.e., Spanish-accented Catalan), which in terms of the Catalan mid-vowels lead to difficulties in the acquisition of the contrast (Bosch and Ramón-Casas, 2011).

Two recent studies examined the production, perception, and processing of the Catalan mid-vowels (/e/-/ε/ and /o/-/ɔ/) by early Spanish-Catalan bilinguals in Majorca. In Amengual (2016), 60 early Spanish-Catalan bilinguals in Majorca completed a categorical AXB discrimination task and picture-naming task to examine the perception and production of the Catalan front and back mid-vowel contrasts. The results showed that the Catalan-specific mid-vowels were more susceptible to discrimination difficulties than other vowel contrasts in the language. Even though these bilinguals were found to maintain robust mid-vowel contrasts in their productions, the degree of language dominance was found to have an effect on the acoustic distance maintained between the mid-vowels. Amengual (2015) explored the perception and processing of these mid-vowels by these same bilingual participants. Results from binary forced-choice identification, AX discrimination, and lexical decision tasks indicated that even though these bilinguals demonstrated a high accuracy in perceptual identification and discrimination tasks, they had difficulties distinguishing between words and non-words in a lexical decision task, with Spanish-dominants exhibiting higher error rates than Catalan-dominants. If cognates are considered to be the crossroads of a bilingual's languages, these “special” lexical items may also be the locus where the bilingual phonologies are more likely to influence each other, affecting a bilingual individual's ability to produce, perceive, and process native-like targets, especially in their non-dominant language.

The present study examines the phonetic production and processing of the Catalan back mid-vowel contrast (/o/-/ɔ/) by 24 highly proficient early Spanish-Catalan bilinguals in Majorca (Spain) that are either Catalan-dominant or Spanish-dominant. Of central importance to this study, the production and lexical decision experiments investigate whether cognate lexical items increase phonetic interference in the acoustic realization and lexical representations of early and highly proficient bilinguals. The amount of overlap in the lexicon depends on the language pair of the bilingual. For instance, with closely related languages such as Spanish and Catalan, the lexicons share many words: between 60 and 85% of the words in the Catalan and Spanish lexicon are cognates (Lewis, 2009; Ramón-Casas et al., 2009). Although the phonological match between cognates in two languages is seldom perfect, correspondences noted between lexical items in two languages have been shown to more likely involve similarities at the phonological level rather than meaning or etymological history (Carroll, 1992). For this purpose, cognate items included as experimental stimuli in this study consist of words that are phonologically, orthographically and semantically similar. Examples of cognate lexical items are Catalan *boca* /bokə/ and Spanish *boca* /boka/ “mouth.” Contrary to the cognate items, the Catalan non-cognate items included in this study are words that do not have an orthographically or phonologically similar translation equivalent in the other language (i.e., Catalan /pɔrk/ and Spanish /θerðo/ “pig”). This is not the first study to examine lexical effects in the production of a Catalan-specific mid-vowel contrast. For instance, cognate effects in the production of the Catalan front mid-vowel contrast (/e/-/ε/) were examined in Mora and Nadeu (2012). The study reports a cognate effect such that the group that used Spanish to a greater extent produced Catalan /ε/ significantly fronter (and thus with F2 values closer to /e/) in cognates than in non-cognates, and there were no significant differences between cognates and non-cognates in terms of vowel height (F1). Vowel height, however, is precisely the dimension that distinguishes Catalan /o/ and /ɔ/ (Recasens and Espinosa, 2006, 2009; Simonet, 2011, 2014).

The main questions that are explored in this study are the following: Is phonetic interference increased in the production of cognates? In other words, does cognate status have an impact on the acoustic realization of these mid-vowels? And also, does cognate status affect the lexical representations of Catalan words that include the Catalan-specific back mid-vowel contrast for these early Spanish-Catalan bilinguals? To the best of my knowledge there are no previous studies that have examined the phonetic production and processing of the same target cognate and non-cognate lexical items in two groups of bilinguals that differ in language dominance. Because of the special status of cognates, it is reasonable to hypothesize that cognates will show different patterns of processing when compared to non-cognates. This cognate effect is expected to extend from the facilitation effects and processing advantages shown in previous psycholinguistic studies, demonstrating a cognate effect on the acoustic production and lexical representations of early bilinguals that affect the ability to maintain native-like contrasts in a language. The present study goes beyond Amengual (2015, 2016), Mora and Nadeu (2012) and Simonet (2011, 2014) in three ways: (i) in comparison to Mora and Nadeu (2012) it examines the phonetic production and processing of a Catalan-specific mid-vowel contrast in Majorcan Catalan, a dialect where the mid-vowels have a different distribution and where a robust contrast may be more available in the ambient input all bilinguals receive, (ii) it investigates the processing abilities of Catalan- and Spanish-dominant bilinguals involving the back mid-vowel contrast (/o/-/ɔ/), thus complementing the production and perception studies on these same back mid-vowels in Simonet (2011, 2014), and (iii) it adds the variable of cognate status to the analysis of these bilinguals' production and processing patterns, a factor that was not examined in Amengual (2015, 2016), in order to better understand the nature of Catalan-Spanish sound system interactions in this group of early and highly proficient bilinguals.

## Experiment 1: Production task

### Method

#### Participants

A total of 24 male Spanish-Catalan bilinguals participated in the production experiment. All participants reported normal speech and hearing and normal or corrected to normal vision, and they all received monetary compensation for their participation in the study. Ages ranged from 18 to 35 (*M* = 21.3, *SD* = 3.42). All participants were born, raised, and educated in Majorca. They reported having extensive exposure to both languages on a daily basis, used Catalan and/or Spanish in the household, and were not native in any other language. This study focuses exclusively on male speakers due to the unbalanced number of male and female participants, which would make it impossible to consider “gender” as a factor if both were to be included.

**Figure 1:**
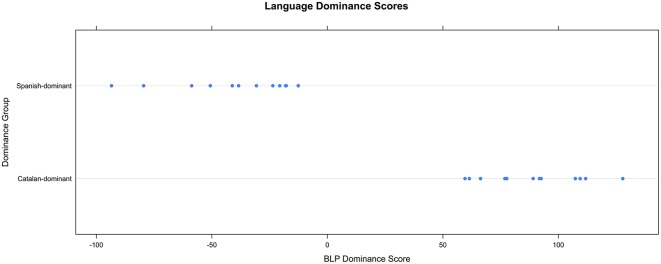
**Language dominance as a function of group according to the BLP (Birdsong et al., 2012)**.

In order to obtain information on the language dominance of the Spanish-Catalan bilingual participants, all participants completed the Bilingual Language Profile (BLP) questionnaire (Birdsong et al., 2012). The BLP is an instrument for assessing language dominance through self-reports and it produces a continuous dominance score and a general bilingual profile taking into account multiple dimensions: age of acquisition of the L1 and L2, frequency and contexts of use, competence in different skills, and attitudes toward each language (see Gertken et al., 2014 for more information). All of these factors are organized in four modules, which received equal weighting in the global language score (language history, language use, language proficiency, and language attitudes). The BLP was administered prior to the production and perception experiments, and was provided in Spanish or Catalan, depending on participant's preference. The classification of participants as Spanish-dominant or Catalan-dominant was determined by the responses to the questionnaire, which generated a global score for each of the languages (Spanish and Catalan), a language particular score for each module, and a global score of dominance. The point system was converted to a scale score with the Catalan score subtracted from the Spanish score. Dominance scores ranged from –93.4 (strongly Spanish-dominant) to 127.8 (strongly Catalan-dominant). Participants with negative points were classified as Spanish-dominant while participants with positive points were classified as Catalan-dominant. Figure 1 provides the distribution of the Spanish- and Catalan-dominant groups.

The main differences between the Catalan-dominant (*N* = 12) and Spanish-dominant (*N* = 12) groups were that Catalan-dominants were exposed earlier to Catalan than Spanish-dominants, the Catalan-dominant group reported a higher daily use of Catalan over Spanish, and also reported a more native-like accent in Catalan in comparison to the Spanish-dominant group. Table 1 provides the language background for each language dominance group.

**Table 1:** **Age, age of exposure, accent self-ratings, and typical daily use of both languages for each language dominance group**.

	**Catalan-dominant**	**Spanish-dominant**
	***M (SD)***	***M (SD)***
Age	21.1 (1.6)	21.5 (4.6)
Age of exposure	CAT = 0 (0)	CAT = 1.8 (2.1)
	SPN = 1.2 (2.3)	SPN = 0 (0)
Self-reported accent (1 = strongly accented; 9 = native-like)	CAT = 8.3 (0.9)	CAT = 6.3 (2.4)
	SPN = 5.3 (1.9)	SPN = 8.5 (0.7)
Typical daily use (1 = only Spanish; 9 = only Catalan)	8.6 (0.8)	3.8 (2.5)

#### Materials

The production of the target Catalan mid-vowels /o/ and /ɔ/ in stressed position for cognate and non-cognate lexical items was elicited in a picture-naming task. The stimuli for this experiment consisted of illustrations representing non-ambiguous objects. Pictorial representations of lexical items were selected instead of the written form to avoid orthographic effects. In order to ensure that the Spanish-Catalan bilingual participants recognized the experimental items as cognates, 10 Spanish-dominant and 10 Catalan-dominant bilinguals that did not participate in the production or lexical decision experiments rated a list of Spanish-Catalan word pairs on a similarity scale (10 = “extremely similar,” 0 = “extremely different”). The ratings for the cross-language pairs were submitted to a one-way ANOVA to ensure that cognate and non-cognate items were rated differently. The analysis confirmed that the ratings for cognate pairs (*M* = 9.25, *SD* = 0.38) and non-cognate pairs (*M* = 2.45, *SD* = 1.46) were significantly different [*F*_(1, 18)_ = 203.22, *p* < 0.001]. The lexical conditions were also matched for word frequency, based on written word frequency in non-literary texts (Rafel i Fontanals, 1998). The lexical frequency of the cognate and non-cognate experimental items with /o/ and /ɔ/ were not significantly different [*F*_(1, 18)_ = 1.99, n.s]. The list of cognate and non-cognate stimuli is included in Table 2.

**Table 2:** **Stimuli included in the production and lexical decision tasks**.

**Catalan**	**Spanish**	**English**	**Target vowel**	**Cognate status**
bota	bota	boot	/o/	Cognate
boca	boca	mouth	/o/	Cognate
ós	oso	bear	/o/	Cognate
copa	copa	glass	/o/	Cognate
doctor	doctor	doctor	/o/	Cognate
flor	flor	flower	/ɔ/	Cognate
escriptori	escritorio	desk	/ɔ/	Cognate
bosc	bosque	forest	/ɔ/	Cognate
sol	sol	sun	/ɔ/	Cognate
pilota	pelota	ball	/ɔ/	Cognate
poma	manzana	apple	/o/	Non-cognate
tassó^*^	vaso	glass	/o/	Non-cognate
tisores	tijeras	scissors	/o/	Non-cognate
papallona	mariposa	butterfly	/o/	Non-cognate
genoll	rodilla	knee	/o/	Non-cognate
porc	cerdo	pig	/ɔ/	Non-cognate
groc	amarillo	yellow	/ɔ/	Non-cognate
taronja	naranja	orange	/ɔ/	Non-cognate
foc	fuego	fire	/ɔ/	Non-cognate
oli	aceite	oil	/ɔ/	Non-cognate

* *Most Catalan-Spanish bilinguals would consider Catalan “tassó” a cognate of Spanish “tazón” (Bowl/Mug) and the Catalan translation of Spanish “vaso” to be “got” (Glass). The translation of the Catalan word “tassó” into Spanish “vaso” is specific to Majorcan Catalan and it is expected that both Catalan- and Spanish- dominant bilingual participants in this study are familiar with this lexical pairing specific to the Majorcan dialect of Catalan*.

#### Procedure

The picture-naming task was conducted individually in a quiet room with participants comfortably seated in front of a computer display. Participants were told that the study involved naming pictures on a computer screen and that their speech would be recorded for subsequent acoustic analysis. All instructions and interactions between the participants and the researcher were in Spanish, independently of participants' language dominance. Spanish, instead of Catalan, was selected as the language to use when giving instructions and interacting with participants because Catalan-dominant bilinguals are generally more comfortable interacting in Spanish than Spanish-dominants are in Catalan. This decision was also made to minimize the potential impact of language mode on bilingual speech behavior, since language mode has been shown to influence the speech production and perception patterns of bilingual individuals (Soares and Grosjean, 1984; Grosjean, 1985, 1997, 1998, 2001, 2008).

Following the instructions in Spanish, participants were presented with the entire set of pictures in randomized order and each picture appeared together with the first letter of the target word. Each picture appeared on a computer screen for 5 s and participants were asked to name the experimental word in Catalan by embedding the target item in a carrier phrase, e.g., “Diuen TARGETWORD cada dia” “(They) say TARGETWORD every day,” and to pronounce as clearly as possible and with a natural pace, speaking neither too quickly nor too slowly. Each session contained four randomized blocks. The Catalan block contained 20 experimental items eliciting the back mid-vowels in Catalan. Because each picture appeared four times (once in each block), each participant produced 80 tokens. A total of 1920 tokens were recorded from the productions of 24 Spanish-Catalan bilinguals. Because six tokens were excluded due to recording errors, or mispronunciations, the dataset comprised a total of 1914 measurements. The speech samples for all participants were recorded using a head-mounted microphone (Shure SM10A) and a solid-state digital recorder (Marantz PMD660), digitized (44 kHz, 16 bit quantization), and computer-edited for subsequent acoustic analysis.

#### Acoustic analysis

Vowels were segmented with *Praat* (Boersma and Weenink, 2015) using synchronized waveform and spectrographic displays. *Praat* scripts were used to parse the recording of each participant into individual files for each target item. The boundaries of each vowel were determined by examining the waveform, spectrogram, and the intensity curve. Formant trajectories, especially the trajectory of the second formant (F2), as well as intensity displays were taken as indicators of vowel onsets and offsets. The onset of the vowel was marked as the beginning of the first voiced cycle where F2 was visible and/or the intensity was similar to that of the vowel's midpoint (for voiceless obstruents), after the release (for voiced stops), the beginning of the first cycle in which F2 was visible and darkened (for fricatives), and at the beginning of the increase in intensity (for nasals and laterals). The end of the vowel was marked by the disappearance of F2, on the last pitch period (before stops and voiceless fricatives), and the beginning of the decline in intensity and the lowering of F2 (before nasals and laterals). When the neighboring segment was an approximant, the onset and offset of the vowel was identified at the beginning of the transitional period between approximant and vowel. Finally for diphthongs, the formant values were calculated at the center-point of the steady-states (i.e., regions of stability with formant differences between time points close to zero) in the target vowel of the two adjacent vowels to avoid transitions. Vowel measurements (F1 and F2) were automatically extracted at the center of the steady-state period of the vowel, together with the duration of the vowel (in milliseconds) using a *Praat* script. Formant tracks were calculated with the Burg algorithm (Anderson, 1974) as built into the *Praat* program. The effective window length for the calculation was set at 25 ms, and was maintained across tokens and speakers. The maximum number of formants to be located by the formant tracker was always 5, and the ceiling was set at 5.0 kHz. Formant values were extracted in Hertz and were further converted to Bark, using the Hz-to-Bark function available in *Praat*. The bark scale is a logarithmic psychoacoustic scale that ranges from 1 to 24, and is a measure of frequency based on the critical bandwidths of hearing believed to reflect human perception (Zwicker, 1961; Traunmüller, 1990; Johnson, 2003). The effects of vocal tract-size differences caused by sex on the acoustics of vowels were minimized because the participant sample consisted exclusively of male speakers. This reduces the need for inter-speaker acoustic normalization procedures (Adank et al., 2004).

### Results

**Figure 2:**
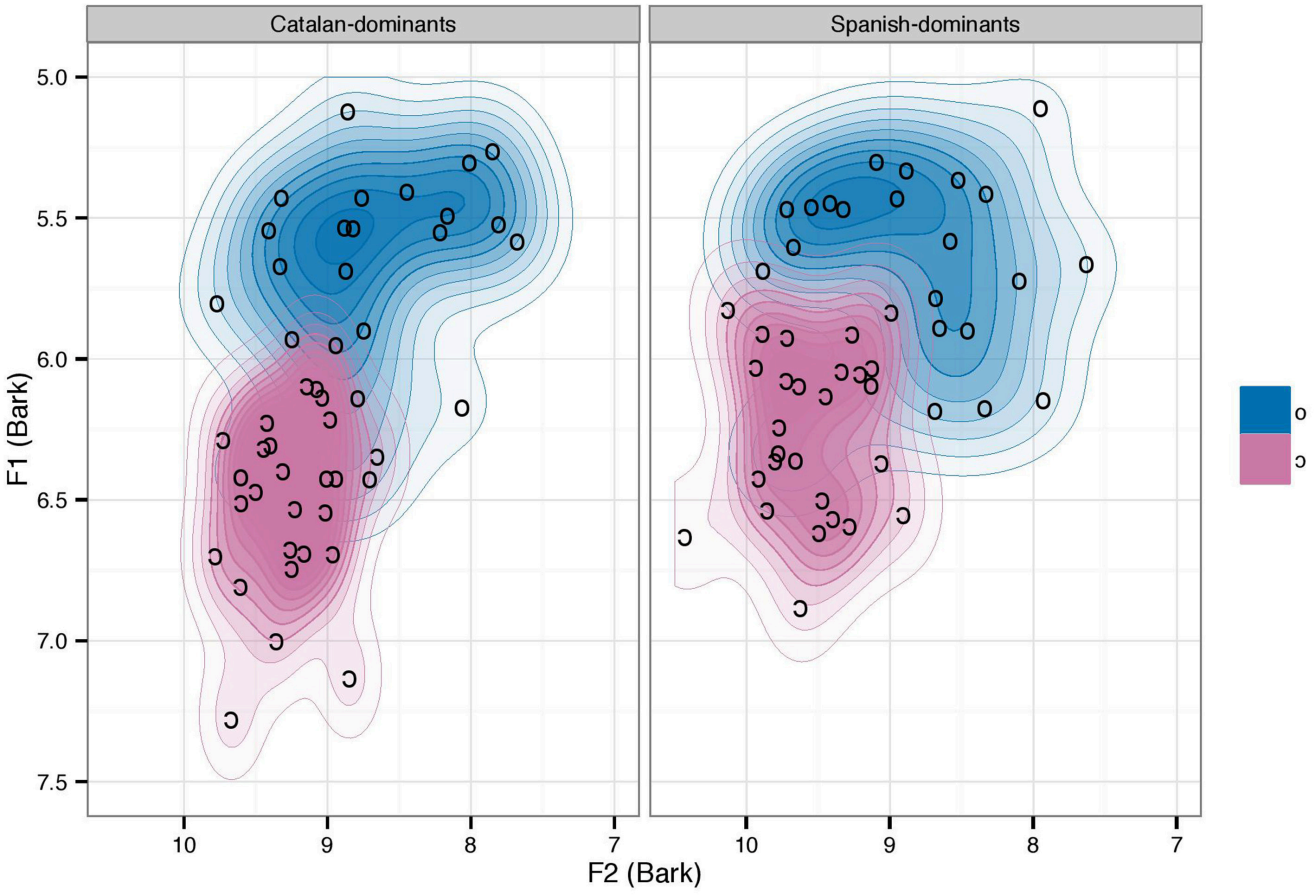
**Bark converted two-dimensional (F1 and F2) contour maps using kernel density estimation plotting the Catalan back mid-vowels as a function of language dominance**.

In order to examine cognate effects in the productions of these bilinguals, datasets of by-subjects aggregates were created including the median F1 and F2 values over subjects as a condition of vowel and cognate status (four values per subject, two per vowel per cognate condition). The dataset was submitted to a mixed-model ANOVA with language dominance (Spanish-dominant, Catalan-dominant) as between-subjects factor, vowel (/o/, /ɔ/), and cognate status (cognate, non-cognate) as within-subjects factors, and subject as the random term. The results on the F1 and F2 data are reported separately below. Figure 2 displays two contour maps plotting the distribution of the Catalan back mid-vowels produced by male Catalan-dominant and Spanish-dominant bilinguals using kernel density estimation (KDE). Inspection of the two-dimensional contour maps shows that both groups maintain the Catalan-specific /o/-/ɔ/ contrast in their productions. This figure also suggests that the back mid-vowel contrast is more robust for Catalan-dominants than for Spanish-dominants, who show more overlap between the /o/ and /ɔ/ acoustic targets.

#### F1 (vowel height)

The mixed-design ANOVA yielded significant main effects of *vowel* [*F*_(1, 22)_ = 110.97, *p* < 0.001] and *cognate status* [*F*_(1, 22)_ = 82.76, *p* < 0.001], but not of *language dominance* [*F*_(1, 22)_ = 2.69, n.s]. In addition, there was a significant interaction between *vowel* and *cognate status* [*F*_(1, 22)_ = 39.44, *p* < 0.001]. No other interactions were significant. The interaction was explored by analyzing the effects of cognate status and language dominance for each vowel separately. Therefore, the dataset was divided into two subsets as a function of vowel. For /o/, the model did not reveal any significant main effects or interactions. For /ɔ/, the analysis yielded a significant effect of *cognate status* [*F*_(1, 22)_ = 145.18, *p* < 0.001] and also an effect of *language dominance* [*F*_(1, 22)_ = 24.39, *p* < 0.001], but there was no significant interaction between *cognate status* and *language dominance* [*F*_(1, 22)_ = 2.24, n.s]. These results indicate that both male Catalan-dominants and Spanish-dominants maintained robust height differences between /o/ and /ɔ/, in such a way that F1 varied as a function of the mid-vowel that was produced. Specifically, /o/ was significantly higher (lower F1 values) than /ɔ/ for both groups. Furthermore, /o/ and /ɔ/ were produced differently in terms of vowel height by each language dominance group and cognate status was found to affect the F1 values of /ɔ/ but not /o/.

#### F2 (vowel fronting)

**Figure 3:**
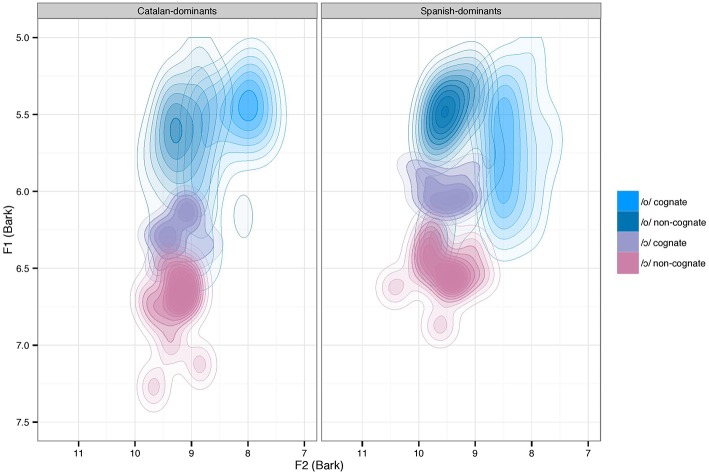
**Bark converted two-dimensional (F1 and F2) contour maps using kernel density estimation plotting the Catalan back mid-vowels as a function of cognate status and language dominance**.

The analysis of F2 revealed a significant main effect of *vowel* [*F*_(1, 22)_ = 85.88, *p* < 0.001] and *cognate status* [*F*_(1, 22)_ = 57.31, *p* < 0.001], and an interaction between *vowel* and *cognate status* [*F*_(1, 22)_ = 48.96, *p* < 0.001], but no effect of *language dominance* [*F*_(1, 22)_ = 3.35, n.s], and no other interactions. The interaction was explored by analyzing the effects of cognate status and language dominance for each vowel separately. Therefore, the dataset was divided into two subsets as a function of vowel. For /o/, the model revealed a significant effect of *cognate status* [*F*_(1, 22)_ = 62.12, *p* < 0.001], but no effect of *language dominance* [*F*_(1, 22)_ = 1.12, n.s], or interaction [*F*_(1, 22)_ = 3.34, n.s]. For /ɔ/, the analysis did not reveal any significant main effects or interactions. These results indicate that /o/ and /ɔ/ differed in F2, but there was no significant difference between the language dominance groups with respect to F2 (fronting). Finally, cognate status was found to affect the F2 values of /o/ but not /ɔ/. Figure 3 displays two contour maps using kernel density estimation (KDE) to plot the Catalan back mid-vowels produced by male Catalan-dominant and Spanish-dominant speakers as a function of cognate status.

**Figure 4:**
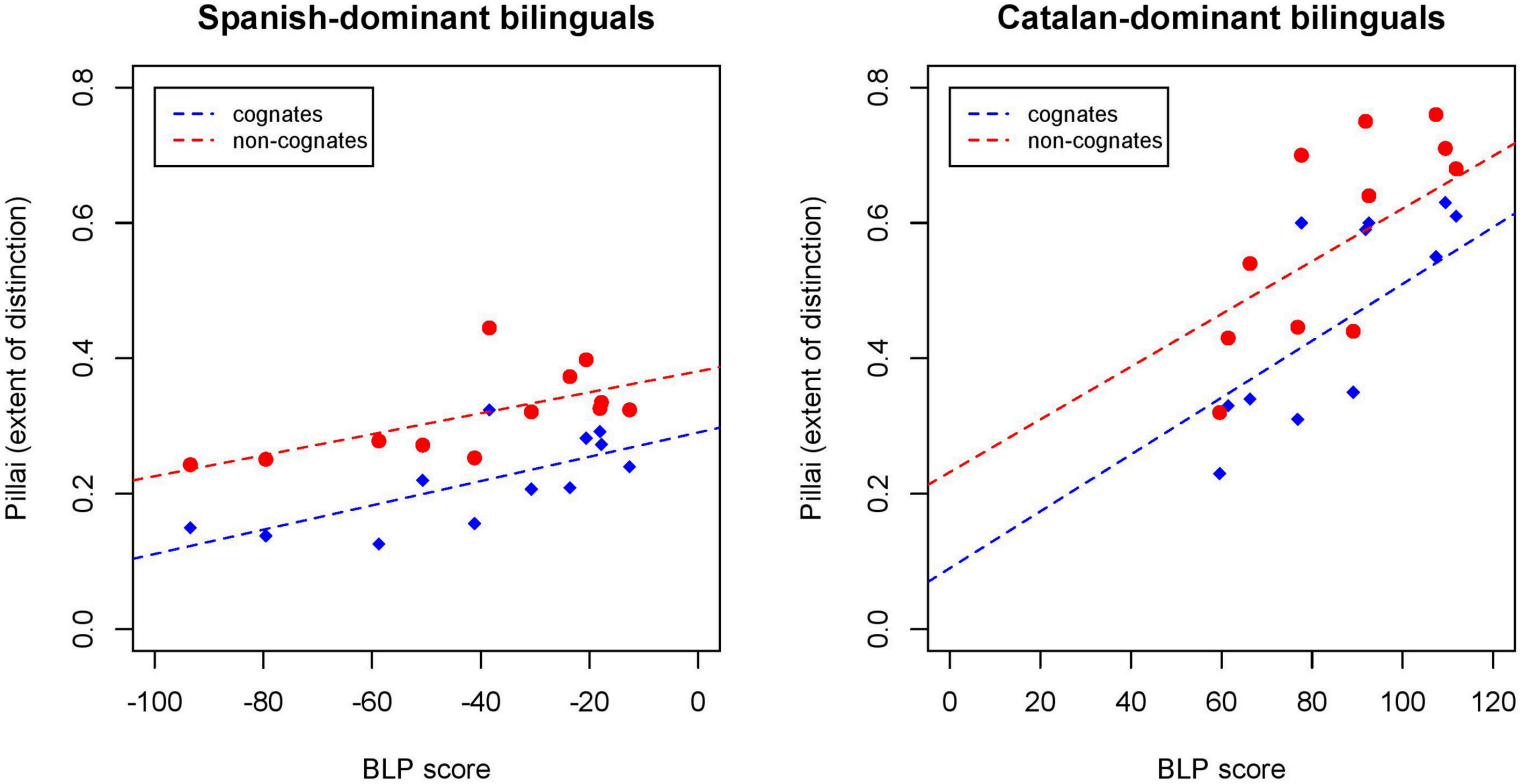
**Individual Pillai scores as a measure of back mid-vowel merger of cognates (blue triangles) and non-cognates (red circles) plotted as a function of a speaker's BLP score**. Fitted lines for cognates (blue) and non-cognates (red).

Because the investigation of group averages often obscures patterns of between-speaker variation, further analyses were carried out to investigate the extent to which the Catalan-specific /o/-/ɔ/ contrast is realized for each individual speaker. The Pillai score is a measure of the degree of merger (Hay et al., 2006; Hall-Lew, 2010; Sloos, 2013). The Pillai score is an output of a Multivariate Analysis of Variance (MANOVA) that represents the degree of overlap between two vowel clusters. In addition to maintaining information about the vowel token cluster distribution, the Pillai score also accounts for phonological environment. The Pillai score representing the vowel cluster difference between /o/-/ɔ/ was calculated for each individual speaker, in which the higher the Pillai score, the lower the degree of overlap, and larger distinction, between the two vowel clusters. As Figure 4 shows, the Pillai score is overall smaller for Spanish-dominant bilinguals (negative BLP score) than for Catalan-dominant bilinguals (positive BLP score), and every participant had a lower Pillai score for cognates (blue triangles) than for non-cognates (red circles). This indicates that each participant produced back mid-vowels with a higher degree of overlap in cognate lexical items. The Pillai value for cognate /o/ and /ɔ/ and for non-cognate /o/ and /ɔ/ in the productions of each individual speaker were correlated with that same speaker's language dominance score. The correlations between language dominance as reported in the BLP and Pillai score of the Spanish-dominant bilinguals showed that there was a significant correlation for cognates (*n* = 12, *df* = 10, *r* = 0.70, *R*^2^ = 0.49, *p* < 0.05) and non-cognates (*n* = 12, *df* = 10, *r* = 0.63, *R*^2^ = 0.40, *p* < 0.05). The analysis of the data from the Catalan-dominant group also revealed that there was a significant positive correlation between the /o/-/ɔ/ Pillai score and the BLP score in the production of cognates (*n* = 12, *df* = 10, *r* = 0.62, *R*^2^ = 0.39, *p* < 0.05) as well as non-cognates (*n* = 12, *df* = 10, *r* = 0.57, *R*^2^ = 0.33, *p* < 0.05). These results show that based on the information provided by the BLP, Spanish-dominants have a higher degree of overlap between these mid-vowels than Catalan-dominants. In addition, the language dominance continuum seems to be a strong predictor of the degree of overlap in the production of the back mid-vowels, as the most Catalan-dominant bilinguals are the ones maintaining a more robust distinction between these mid-vowels.

## Experiment 2: Lexical decision task

### Method

#### Participants

Participants were the same Spanish-Catalan bilinguals that participated in Experiment 1.

#### Materials

The experimental stimuli for the lexical decision task consisted of the same list of 20 Catalan words used in the production experiment. The Catalan experimental items, which either contained the target mid-vowel /o/ or /ɔ/ in stressed position, were matched in word frequency and were further divided into cognate and non-cognate words according to similarity ratings (see Materials). The corresponding incorrectly pronounced words (i.e., non-words) were created by replacing the stressed mid-vowel with the other member of the contrast for each lexical item. For instance, the Catalan non-word ^*^/bosk/ was created from the real word /bɔsk/ “forest.” Conversely, the correct pronunciation of /bokə/ “mouth” appeared alongside ^*^/bɔkə/ in the stimuli list. The complete list of experimental stimuli is presented in Table 3.

**Table 3:** **Experimental items used in the lexical decision task**.

**/o/-/ɔ/**				**Cognate status**
**/o/ word type**		**/ɔ/ word type**		
**Word**	**Non-word**	**Word**	**Non-word**	
***/o/ → /o/***	/o/ → ^*^/ɔ/	***/ɔ/ → /ɔ/***	/ɔ/ → ^*^/o/	
/botə/	^*^/bɔtə/	/flɔ/	^*^/flo/	Cognate
/bokə/	^*^/bɔkə/	/əskriptɔri/	^*^/əskriptori/	Cognate
/os/	^*^/ɔs/	/bɔsk/	^*^/bosk/	Cognate
/kopə/	^*^/kɔpə/	/sɔl/	^*^/sol/	Cognate
/dokto/	^*^/doktɔ/	/pilɔtə/	^*^/pilotə/	Cognate
/pomə/	^*^/pɔmə/	/pɔrk/	^*^/pork/	Non-cognate
/təso/	^*^/təsɔ/	/grɔk/	^*^/grok/	Non-cognate
/tizorəs/	^*^/tizɔrəs/	/tərɔn$\hat{d_{ʒ}}$ə/	^*^/təron$\hat{d_{ʒ}}$ə/	Non-cognate
/pəpəʎonə/	^*^/pəpəʎɔnə/	/fɔk/	^*^/fok/	Non-cognate
/$\hat{d_{ʒ}}$ənoʎ/	^*^/$\hat{d_{ʒ}}$ənɔʎ/	/ɔli/	^*^/oli/	Non-cognate

* *indicates the incorrect mid-vowel (non-word)*.

The auditory stimuli presented in the lexical decision task were obtained from the productions of three male native Majorcan Catalan speakers. The native speakers were asked to clearly enunciate the 40 experimental words (20 words and 20 non-words) providing 10 repetitions of each lexical item. The recordings of the words and non-words were made using a Shure SM10A dynamic head-mounted microphone and a solid-state digital recorder (Marantz PMD660), and digitized at 44 KHz and 16 bits. In order to select the best “exemplars” for each word and non-word, three separate datasets (one for each speaker) were created including the median F1 and F2 values for each lexical item as a condition of vowel and vowel status (correct/incorrect). To ensure that there were only significant differences between /o/ and /ɔ/ productions independently of vowel status, each subset was submitted to a repeated measures ANOVA with F1 as the dependent variable, vowel (two levels: /o/ and /ɔ/) and vowel status (two levels: correct and incorrect). After confirming that the tokens selected based on the F1 median differed with respect to the vowel, but not because of vowel status (e.g., a mispronounced /ɔ/ vowel was not significantly different from a correctly pronounced /ɔ/ word), the same dataset was submitted to a repeated measures ANOVA with F2 (Hz) as the dependent variable, and with vowel and vowel status as the independent variables. The statistical analyses again supported the initial selection of the median F1 as a measure to select the best exemplar of a word and non-word for each speaker. To summarize, the stimuli selected contained lexical items in which a properly pronounced /o/ was not different in height (F1) or fronting (F2) to a mispronounced target item produced with /o/ for any of the three speakers. The stimuli were normalized for peak intensity. If there was a DC offset, it was removed and the maximum amplitude was normalized to −0.5 dB at a project rate of 44 KHz. The picture stimuli that were presented together with the auditory stimuli consisted of the same pictorial representations employed in the picture-naming task.

#### Procedure

Participants completed the lexical decision task seated comfortably in front of a computer screen, and the stimulus presentation software *SuperLab 4.5* (Cedrus Corporation, USA) controlled the presentation of visual and auditory stimuli. Participants were told that the stimuli would consist of words and non-words, and that non-words were based on real words but with the stressed vowel changed (e.g., /o/ to /ɔ/, and vice versa). Participants were asked to classify each stimulus as being either a word or a non-word by pressing the right button on the USB Response Pad (RB-730) immediately after hearing a word stimulus, and the left button on hearing a non-word. The identity of the buttons was counterbalanced between subjects and the order of presentation was randomized for each participant. Participants responded to a total of 122 trials: 2 practice trials + 120 randomized test trials. Specifically, the experimental data consisted of 20 tokens × 2 type (correct/incorrect) × 3 voices = 120 responses per participant. As there were 24 participants, the dataset was comprised of 2880 data points.

### Results

The lexical decision data were analyzed in a series of mixed-design ANOVAs, with language dominance (Spanish-dominant, Catalan-dominant) as between-subjects factor, vowel (/o/, /ɔ/) and cognate status (cognate, non-cognate) as within-subjects factor, and participant as the random term. The results for words and non-words are presented separately in order to analyze how Spanish-dominant and Catalan-dominant bilinguals differ in their categorization of mispronounced and properly pronounced words that vary exclusively in the Catalan back mid-vowel contrast. For this purpose, two datasets were created: the first one consisting of the responses to correctly produced real words, and the second one only including the responses to mispronounced words (i.e., non-words). The error rate (%) and response time data (ms) obtained from stimulus onset are presented for words and non-words.

#### Error rate: Properly pronounced words (/o/ → /o/ and /ɔ/ → /ɔ/)

The analysis of the correctly produced /o/ and /ɔ/ stimuli did not yield significant main effects of *language dominance* [*F*_(1, 22)_ = 0.29, n.s], *cognate status* [*F*_(1, 22)_ = 0.14, n.s] or *vowel* [*F*_(1, 22)_ = 0.70, n.s]. The model, however, did reveal a significant interaction between *vowel* and *cognate status* [*F*_(1, 22)_ = 55.16, *p* < 0.001]. The interaction between vowel and cognate status was explored by analyzing the effects of cognate separately for each vowel. Bonferroni-corrected paired t-tests confirmed that there were significant differences in the categorization accuracy of these bilinguals between cognates and non-cognates in /o/ type words [diff. = –6.10, *t*_(23)_ = –5.78, *p* < 0.001], and also in /ɔ/ type words [diff. = 6.66, *t*_(23)_ = 5.53, *p* < 0.001]. These results confirm that when responding to properly pronounced words these bilinguals made more mistakes in non-cognate than in cognate /o/ type words, but the effect was in the opposite direction in /ɔ/ type words: cognates elicited a higher error rate than non-cognates.

#### Error rate: Non-words (/o/ → ^*^/ɔ/ and /ɔ/ → ^*^/o/)

**Figure 5:**
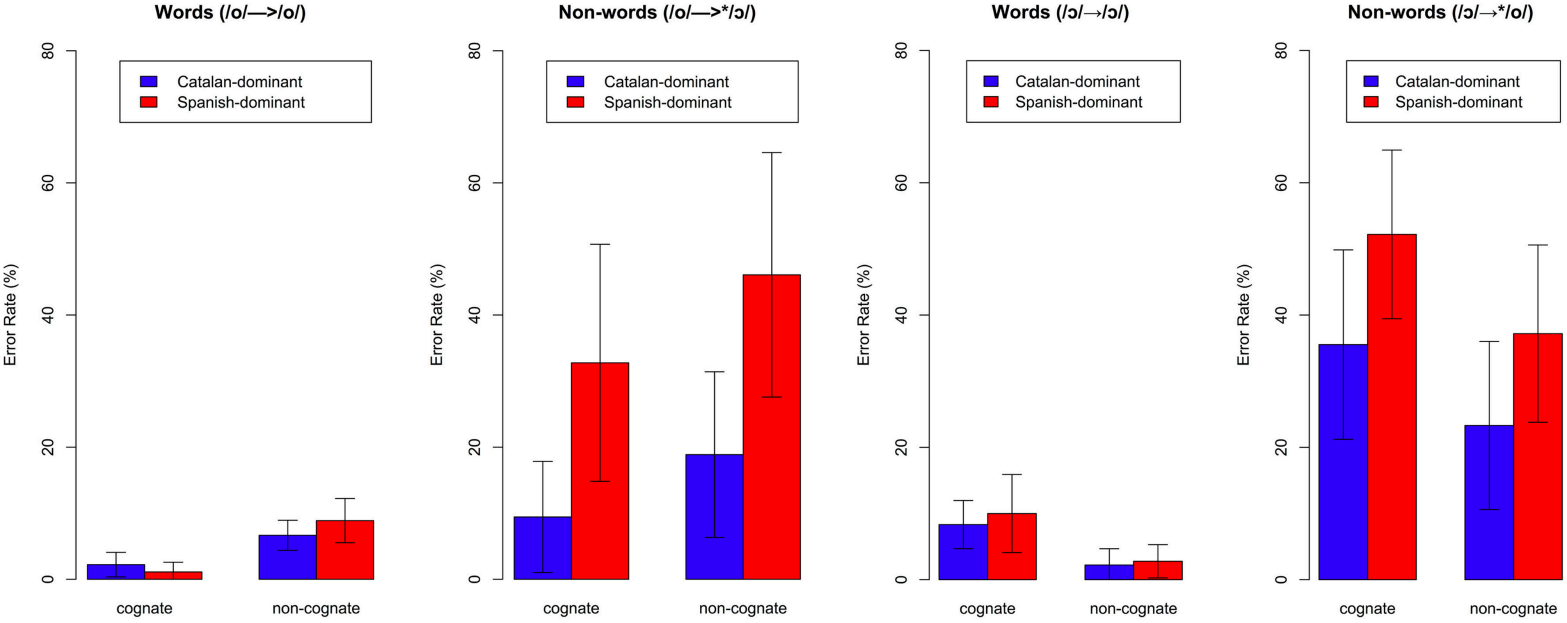
**Error rate (%) for cognate and non-cognate items as a function of vowel type (/o/, /ɔ/) and vowel status (word, non-word) by language dominance**. Error bars enclose ± one standard error.

The analysis of the non-words revealed significant main effects of *language dominance* [*F*_(1, 22)_ = 5.16, *p* < 0.05] and *vowel* [*F*_(1, 22)_ = 5.10, *p* < 0.05], but the model did not yield a significant effect of *cognate status* [*F*_(1, 22)_ = 1.59, n.s]. However, there was a significant interaction between *vowel* and *cognate status* [*F*_(1, 22)_ = 49.92, *p* < 0.001]. This interaction was explored by analyzing the effects of cognate separately for /o/ → ^*^/ɔ/ and /ɔ/ → ^*^/o/. Bonferroni-corrected paired *t*-tests confirmed that there were significant differences in the error rate between cognates and non-cognates in /ɔ/ → ^*^/o/ [diff. = −11.38, *t*_(23)_ = −5.05, *p* < 0.001], and also in /o/ → ^*^/ɔ/ [diff. = 13.61, *t*_(23)_ = 8.35, *p* < 0.001]. These results indicate that Spanish-dominant and Catalan-dominant bilinguals differed in their categorization of non-words in the lexical decision task. Spanish-dominant bilinguals in particular had great difficulties in recognizing mispronounced words that differed in the back mid-vowel contrast. Furthermore, cognate status was found to affect the categorization of /ɔ/ words incorrectly pronounced as /o/, and also /o/ words mispronounced as /ɔ/, but having an effect on the opposite direction. Cognates in /ɔ/ words incorrectly pronounced as /o/ showed a higher error rate than non-cognates indicating that having a cognate in Spanish with /o/ created more interference causing a higher proportion of non-words accepted as real words. In the case of /o/ words mispronounced as /ɔ/, the pattern showed that cognates elicited a lower error rate than non-cognates. Figure 5 shows the error rate (%) in the categorization of words and non-words for each back mid-vowel as a function of cognate status, vowel status and language dominance.

#### Response times

**Figure 6:**
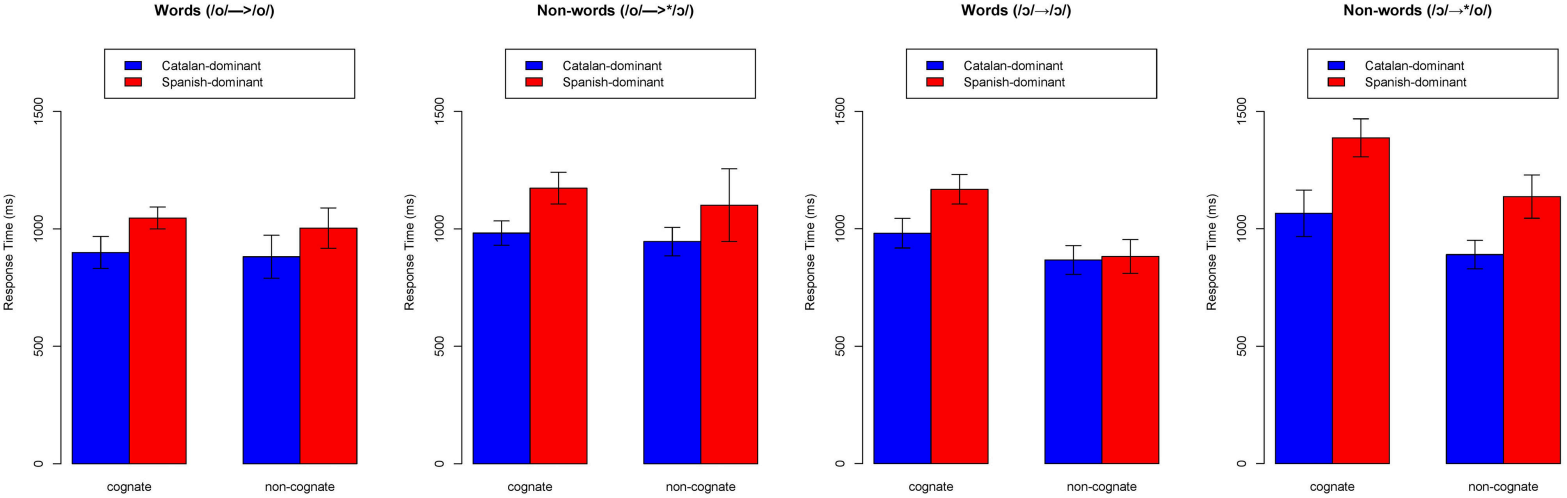
**Response times (ms) for cognate and non-cognate items as a function of vowel type (/o/, /ɔ/) and vowel status (word, non-word) by language dominance**. Error bars enclose ± one standard error.

A dataset that included the median response times (ms) over subjects as a condition of vowel (/o/, /ɔ/) and word status (correct, incorrect) was created (four values per subject). The median response times were calculated over accurate trials only, and a non-response was recorded if the participant did not press a key in the 2-s interval allowed. There were a total of 9 non-responses that were removed from the dataset. This dataset was submitted to a mixed-model ANOVA with language dominance (Catalan-dominant, Spanish-dominant) as between-subjects factor, vowel (/o/, /ɔ/), word status (correct, incorrect), cognate status (cognate, non-cognate) as within-subjects factors, and participant as the random term. The model yielded significant main effects of *language dominance* [*F*_(1, 22)_ = 20.53, *p* < 0.001], *vowel* [*F*_(1, 22)_ = 8.60, *p* < 0.01], *cognate status* [*F*_(1, 22)_ = 38.30, *p* < 0.001], and *word status* [*F*_(1, 22)_ = 107.42, *p* < 0.001]. In addition, there was a significant interactions between *vowel* and *cognate status* [*F*_(1, 22)_ = 36.19, *p* < 0.001]. The significant interaction was explored by analyzing the effects of cognate status for each vowel separately. For the /o/ type stimuli (*(/o/*→*/o/* and */o/*→^*^/ɔ*/*), the model revealed a significant effect of *language dominance* [*F*_(1, 22)_ = 11.58, *p* < 0.001] and *word status* [*F*_(1, 44)_ = 20.34, *p* < 0.001], but no effects of *cognate status* [*F*_(1, 22)_ = 2.10, n.s]. For the /ɔ/ type stimuli (*/*ɔ*/*→*/*ɔ*/, /*ɔ*/* → ^*^/o/), there was a significant effect of *language dominance* [*F*_(1, 22)_ = 28.22, *p* < 0.001], *cognate status* [*F*_(1, 22)_ = 130.6, *p* < 0.001], *word status* [*F*_(1, 44)_ = 32.22, *p* < 0.001], and significant interactions between *language dominance* and *cognate status* [*F*_(1, 22)_ = 11.7, *p* < 0.001] and between *language dominance* and *word status* [*F*_(1, 44)_ = 12.78, *p* < 0.001]. These results show that Spanish-dominants took longer to respond to words and non-words that differed in the back mid-vowel contrast in comparison to Catalan-dominants. In addition, both groups had longer reaction times when responding to non-words than to real words. Finally, cognate effects were found in the response times of the /ɔ/ type stimuli, but these effects were not noticeable in the response times of the /o/ type words for both groups. Figure 6 provides the response times (ms) as a function of vowel and cognate status for each language dominance group.

**Figure 7:**
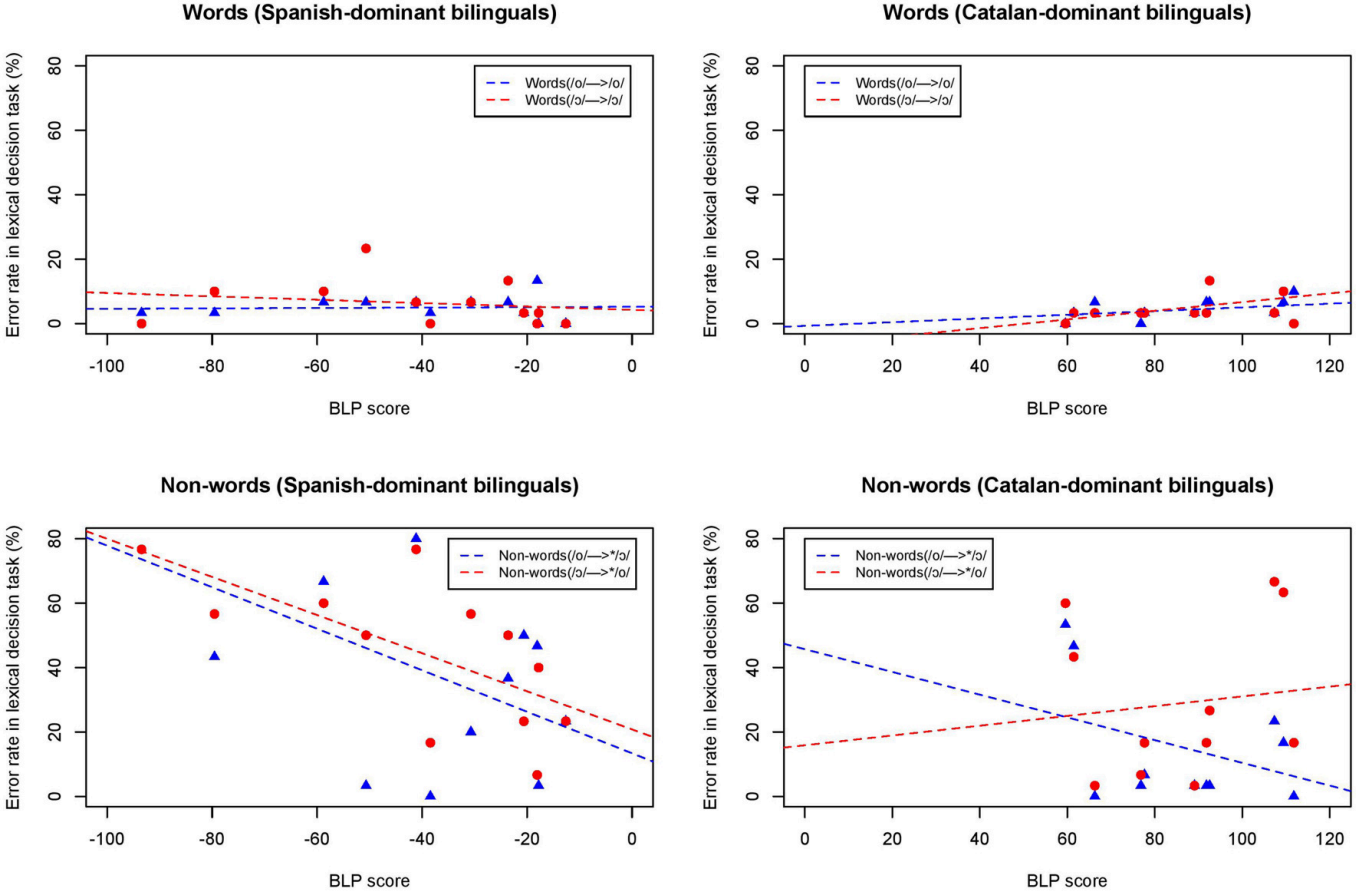
**Individual error rates (%) for words and non-words plotted as a function of a speaker's BLP score**. Fitted lines for /o/-type words (blue) and /ɔ/-type words (red).

In order to investigate individual variation in the lexical decision task, the average error rate for words and non-words was calculated separately for each individual participant. The individual error rate (%) in the lexical decision task was correlated with the participants' language dominance score as reported in the BLP. As Figure 7 shows, the error rates are in general higher for Spanish-dominant bilinguals (negative BLP score) than for Catalan-dominant bilinguals (positive BLP score), and also both groups display a much higher error rate when responding to non-words than to correctly pronounced words. The correlations between BLP score and error rate for words and non-words as a function of language dominance are presented in Table 4.

**Table 4:** **Results from the correlations between BLP score and error rate for words and non-words**.

**Stimulus**	**Spanish-dominant bilinguals**	**Catalan-dominant bilinguals**
Words *(/o/ → /o/*)	*n* = 12, *df* = 10, *r* = 0.04, *R*^2^ = 0.002, n.s	*n* = 12, *df* = 10, *r* = 0.42, *R*^2^ = 0.17, n.s
Words *(/*ɔ*/ → /*ɔ*/*)	*n* = 12, *df* = 10, *r* = −0.19, *R*^2^ = 0.03, n.s	*n* = 12, *df* = 10, *r* = 0.56, *R*^2^ = 0.25, n.s
Non-words (/o/ → ^*^/ɔ*/*)	*n* = 12, *df* = 10, *r* = −0.52, *R*^2^ = 0.27, *p* < 0.05	*n* = 12, *df* = 10, *r* = −0.42, *R*^2^ = 0.17, n.s
/ɔ/-type (*/*ɔ/ → ^*^/o/)	*n* = 12, *df* = 10, *r* = −0.66, *R*^2^ = 0.44, *p* < 0.01	*n* = 12, *df* = 10, *r* = 0.14, *R*^2^ = 0.01, n.s

The correlations between BLP score and error rate in the lexical decision task revealed that there was not a significant correlation for the Catalan-dominant-dominant or Spanish-dominant group in any of the stimuli, except for a significant correlation for the Spanish-dominants responding to both types of non-words *(/o/*→^*^/ɔ*/ and /*ɔ*/*→^*^/o/). These results show that there was a higher error rate in the lexical decision task as a function of being more Spanish-dominant, but this was only the case when responding to non-words. Further analyses also determined that there was not a significant correlation between the response time data with the error rate, that is, individuals who were faster at responding did not necessarily obtain lower or higher error rates.

**Figure 8:**
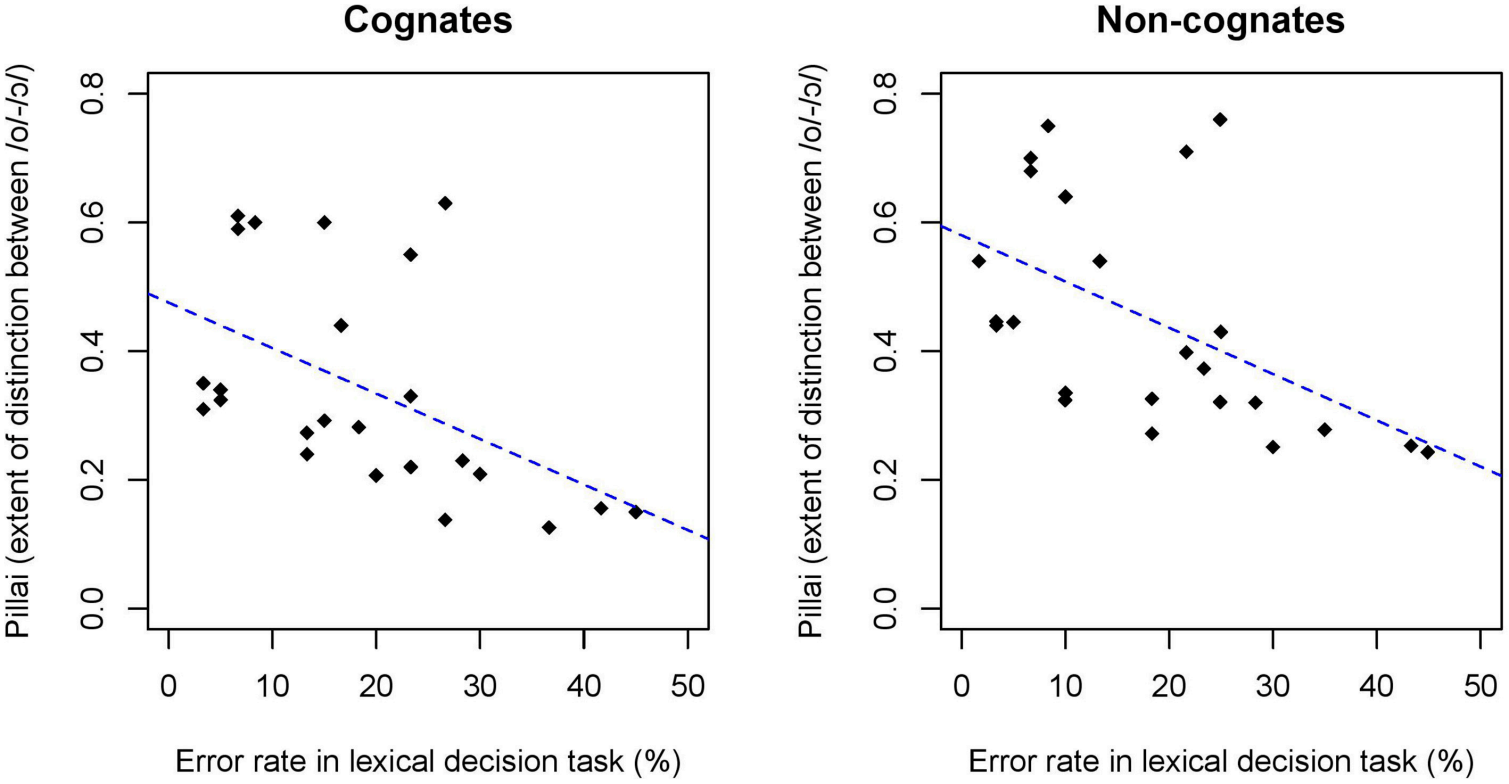
**Accuracy rate in the lexical decision task plotted as a function of the Pillai score of each individual speaker by cognate status**. Fitted lines for cognates (left) and non-cognates (right).

The relationship between the speech production and perception of these early bilinguals was also examined. The Pillai scores of each individual speaker were compared to their error rates in the lexical decision task, collapsing words and non-words, for both cognates and non-cognates. The analyses revealed that there was a significant correlation between the Pillai score and accuracy in the lexical decision task for cognates (*n* = 24, *df* = 22, *r* = −0.50, *R*^2^ = 0.25, *p* < 0.05) and non-cognates (*n* = 24, *df* = 22, *r* = −0.51, *R*^2^ = 0.26, *p* < 0.05]. Figure 8 plots the accuracy rate in the lexical decision task and the individual speaker's Pillai score between /o/ and /ɔ/ as a function of cognate status. These results indicate that there is a correlation between the production of the back mid-vowel contrast and the ability to recognize properly pronounced and mispronounced words: bilinguals who produced the Catalan back mid-vowel contrast with a higher degree of overlap (i.e., smaller Pillai score) were more likely to have a higher error rate when responding to cognates and non-cognates in the lexical decision task.

## Discussion

Everyday linguistic performance involves much more than the ability to concentrate on isolated phonetic segments in speech perception and production experiments. In human communication, a combination of sounds are necessarily embedded in words, so beyond the ability to discriminate stimuli and produce acoustic targets, speakers must also encode these language-specific phonemes in the form of spoken words in their mental lexicon. Therefore, a language user must seamlessly learn which combination of vowel and consonant units are contained in a given word, and also be able to recognize which words include a specific phonemic category. Spanish-Catalan bilinguals must acquire two vowel systems with a different set of segments, and crucially, they must learn to select the correct vowel depending on the lexical item that is going to be pronounced. This study probes if Spanish-Catalan bilinguals are able to produce and recognize the appropriate Catalan-specific mid-vowel in lexical items in general, and if cognates in particular enhance cross-linguistic influence.

The present study investigated cognate effects in a picture-naming and lexical decision task on the Catalan back mid-vowel contrast (/o/-/ɔ/) by 12 Spanish-dominant and 12 Catalan-dominant male Spanish-Catalan bilinguals from Majorca (Spain), complementing the findings from previous studies in the same bilingual setting (Amengual, 2015, 2016). These early and highly proficient bilinguals have been raised in a bilingual community where they have been exposed to both Catalan and Spanish before the age of 4. The results from recent studies in Majorca, and contrary to previous findings in Barcelona, indicate that both Spanish-dominants and Catalan-dominants maintain robust mid-vowel contrasts in their productions and also demonstrate high perceptual accuracy when completing identification, AX discrimination, and AXB discrimination tasks. However, even though these bilinguals perform at ceiling in the perceptual tasks that consist of identifying and discriminating between isolated phonemes, their performance decreases in the lexical decision task. This is consistent with previous research showing that even high accuracy in phonetic categorization will not guarantee accurate lexical encoding of a difficult L2 contrast (Darcy et al., 2013). Adding to the previous literature, this study posed a different question regarding the phonetic production and processing abilities of these early bilinguals: Do cognates increase phonetic interference in the acoustic realization and lexical representations of these bilinguals? To answer this question, cognates and non-cognates were examined to detect cross-language influence. Non-cognates such as Catalan *poma* /pomə/ “apple” (Spanish *manzana* /manθana/) were investigated alongside cognates, such as *bosc* /bɔsk/ “forest” (Spanish *bosque* /boske/).

The results of the picture-naming and lexical decision tasks provide evidence of cognate effects in both the phonetic production and processing of the Catalan back mid-vowel contrast. This cross-linguistic influence was robust for both language dominance groups when selecting the appropriate phonetic representations of lexical items in order to produce the experimental stimuli as well as when identifying aurally presented stimuli either as a word or a non-word. Cognate status was found to influence both the vowel height and fronting for the Catalan back mid-vowels /o/ and /ɔ/ in the productions of both Spanish-dominant and Catalan-dominant bilinguals. The cognate status effect was especially robust in the production of the Catalan-specific /ɔ/. The production data showed that /ɔ/ in cognate lexical items were produced significantly higher than non-cognates, approximating the /o/ acoustic region. In other words, the cognate items were taking a different direction than non-cognates, reducing the acoustic distance between /o/ and /ɔ/. Further evidence of phonetic interference at the lexical level was found in the lexical decision task. Results show that when responding to cognates in /ɔ/words incorrectly pronounced as /o/ there was an increased cross-linguistic interference between the mid-vowel categories causing a higher error rate and longer response times. In this case there was a higher proportion of non-words accepted as real words. The opposite effect was found in the case of /o/ words mispronounced as /ɔ/. In this case, the pattern showed that cognates increased lexical decision accuracy in comparison with non-cognates. Taken together these results suggest that congruent cognates (cognates that contain a stressed mid-vowel in Spanish and a higher-mid vowel in Catalan, i.e., /o/-/o/) increased the lexical decision accuracy, facilitating lexical access, whereas incongruent cognates (cognates that contain a stressed mid-vowel in Spanish and a lower-mid vowel in Catalan, i.e., /o/-/ɔ/) increased cross-linguistic interference between the mid-vowel categories, causing a higher error rate in the lexical recognition process. The results from the reaction time data also show an effect of language dominance and word type: Spanish-dominant bilinguals took longer to respond to the stimuli than Catalan-dominants and both groups had a longer response latency with non-words (i.e., lexical items based on real words, but with the alternate mid-vowel pair) than real words. Finally, both groups took longer to respond to cognates in the /ɔ/ type stimuli, but these effects were not noticeable in the response times of the /o/ type words.

Analyses of individual data showed that the degree of language dominance as a function of a participant's BLP score had an effect on the error rate in the lexical decision task. Specifically, those participants that were more Spanish-dominant were the ones that were most likely to have a higher error rate when responding non-words. Similarly, the degree of language dominance was a strong predictor of the acoustic distance and overlap maintained between both phonemes. The Pillai score, which measures the degree of merger between two vowel clusters significantly correlated with the degree of language dominance. For Spanish-dominants there was a significant correlation between the degree of overlap of the /o/-/ɔ/ and the degree of Spanish dominance, as operationalized by the BLP. Similarly, for the Catalan-dominant group there was a more robust distinction between the back mid-vowels as a function of being more Catalan-dominant. Cognate effects were also evident in the individual data, as both Catalan-dominants and Spanish-dominants produced /o/ and /ɔ/ with a higher degree of overlap (i.e., lower Pillai score) in cognate than in non-cognate lexical items. Finally, the present study also examined the relationship between the phonetic production and perception abilities of each bilingual individual. The correlations between the production and lexical decision data indicate that there is a tight link between the production of the back mid-vowel contrast and the ability to recognize properly pronounced or mispronounced cognates and non-cognates in a lexical decision task. These findings provide evidence that cross-language phonetic interference occurs when early Spanish-Catalan bilinguals access their mental lexicon. The acoustic properties of cognate lexical items result in phonetic alterations in the lexical representations of these bilingual individuals.

Such an effect must be operationalized in a model of the bilingual lexicon that accounts for the variable production and lexical decision patterns linked to the bilinguals' lexical representations. The Perceptual Assimilation Model (PAM; Best, 1995), Perceptual Assimilation Model of Second Language Speech Learning (PAM-L2; Best and Tyler, 2007), and the Speech Learning Model (SLM; Flege, 1995) are models of cross-linguistic speech perception and production that assume that the learnability of new sounds in the L2 is perceptual in nature and depends on the perceived phonetic distance between the sounds in the L2 and the most similar segments in the L1 phonetic inventory. Despite these common assumptions, these models address different aspects of L2 phonological acquisition: the SLM focuses on individual phonetic categories whereas the PAM and PAM-L2 focus on pairwise phonological contrasts, and the SLM was primarily designed to address L2 production, whereas the PAM and PAM-L2 have a main focus on non-native speech perception and L2 perception respectively. The SLM, PAM, and PAM-L2 make straightforward predictions about the learnability of L2 sounds depending on the perceived similarity between the sounds of the L1 and L2. However, these models cannot account for an interaction between the phonological and lexical levels of representation across the two languages of a bilingual individual. In other words, these models cannot predict the phonetic interference found in the production and lexical decision of cognate lexical items, nor how the acoustic characteristics of the Catalan mid-vowels are related to the lexical representations stored in the bilingual mental lexicon. How can these results be theoretically interpreted?

Cognate facilitation effects in bilingual speech production have previously been explained with spreading activation models of speech production, such as cascaded activation models of lexical access (Dell, 1986; Goldrick and Blumenstein, 2006), in opposition to a strictly discrete activation model (Levelt, 1989; Levelt et al., 1999). Crucially, the differences between these theoretical approaches are that the discrete models would not predict that lexical variables such as cognate status could affect its phonetic realization, because in this view, selection is made at the lexical level before articulation. As a result sublexical representations become active only after the target word has been selected. The cascaded activation models propose that processes at the lexical and phonological levels of planning can cascade down to affect the articulatory realization of acoustic targets. For instance, Jacobs et al. (2016) investigated effects of cross-language activation in the productions of L2 Spanish speakers of differing proficiencies (highly proficient speakers, intermediate learners in a domestic immersion program, and intermediate speakers in a classroom setting). Because the results from their study show effects of cognate status only in the articulation of the intermediate classroom learners of Spanish but not with the other groups, the authors argue that the speech production system of these bilinguals is cascaded, but that it exhibits “staged vs. cascading behavior as a function of task difficulty” (Jacobs et al., 2016, p. 25). A recent study, however, questions the cascading nature of the planning system. Buz and Jaeger (2016), using a picture-naming experiment, investigate the effects of phonological neighborhood density and provide evidence that the effect of phonological neighborhood density on word duration and vowel dispersion does not seem to be mediated through lexical planning (Buz and Jaeger, 2016), but admit that word-specific phonetic representations are compatible with their findings.

Assuming that lexicons in different languages are mentally interconnected (Costa et al., 2005; Jarvis and Pavlenko, 2008), lexical representations in one language are predicted to affect the lexical representations in the other. Exemplar models of lexical representation (Goldinger, 1997, 1998; Johnson, 1997a,b; Bybee, 2001; Pierrehumbert, 2001, 2003a,b; Hawkins, 2003) are theoretic approaches that are able to explore the lexical/phonetic interface in which the mental lexicon is represented phonetically. For the purpose of this study, the model is expanded to include bilingual data in order to analyze the interactions between the lexical representations of both languages in the bilingual lexicon. Adapting the exemplar model to bilingual lexicons can account for the interaction between the phonological and lexical levels of representation across a bilingual's languages and can explain the findings in the Majorcan bilingual phonetic production and processing of cognates and non-cognates.

Exemplar models assume that speech perception and production are closely linked. Clusters of similar experiences—that is, “exemplars” of the same word—are formed including productions that share a particular acoustic property. These exemplars are categorized by their similarity to extant stored exemplars so that clouds of memory traces group similar exemplars close to each other while dissimilar ones are more distant. The exemplars themselves include much more than just purely phonetic information: the representation of a specific word includes its meaning(s) and all the acoustic, lexical, social, and contextual information from the perceptual event (Ettlinger and Johnson, 2009). Exemplar models assume that when a new stimulus is presented, the memory traces (i.e., exemplars) are activated in proportion to their similarity to the stimulus, and the pattern of activation is used to determine the category membership of the exemplar. This automatically eliminates a separation between pre-lexical and lexical phonological processing abilities (Mehler, 1981; McClelland and Elman, 1986; Pisoni and Luce, 1987; Norris, 1994; Gaskell and Marslen-Wilson, 1997). Such a model accounts for how speakers might possess fine-grained, detailed, and word-specific knowledge about the sounds and words of their language and require no phonological abstraction prior to lexical access (Pierrehumbert, 2001; Coleman, 2002; Johnson, 2007).

The application of an exemplar-based approach to the production and perception of early Spanish-Catalan bilinguals might assume mostly distinct exemplar clouds representing Catalan and Spanish. However, since these clouds are organized by the phonetic similarity of the exemplars and also include semantics, there is likely to be an overlap between the two otherwise independent language systems with respect to cognates. Since cognates by their very nature have the same meaning and similar phonetic forms in the two languages, the exemplar clouds for such cross-linguistic pairs (e.g., Catalan /sɔl/ “sun” and Spanish /sol/ “sun”) may in fact overlap, such that exemplars from both languages exist in the same perceptual space. Thus, bilingual production and lexical decision of cognates potentially draws from both Catalan and Spanish exemplars instead of restricting the possible targets to the language-specific exemplars available for each language separately.

The results reported in this study indicate that the cognate status of a lexical item influences the production targets and the selection of the correct phonetic category in a lexical decision task. In the picture-naming task, the phonetic output of a specific lexical item of a Spanish-Catalan bilingual is the average over the set of exemplars in the vicinity of a randomly selected exemplar. Therefore, cognate effects would result from the selection of a region in the exemplar space, and specifically the average over this region containing overlapping acoustic properties. For example, the acoustic properties of the target word /sɔl/ “sun” might be influenced by the average over the region in the exemplar space that contains instances of /sol/ exemplars from Spanish, as opposed to the Catalan word /ɔli/ “oil,” where the average from the exemplar space would not be affected by the acoustic properties of Spanish exemplars in the cloud of memory traces containing a back mid-vowel (Spanish *aceite* /aθeite/). In other words, a cognate effect in production is expected if the average over a cloud of memory traces in the exemplar space includes instances of Spanish-influenced exemplars (i.e., Spanish words or Spanish-accented Catalan words) instead of native-like Catalan exemplars, ultimately having an impact on the acoustic realization of this Catalan-specific vowel contrast. The average over a region in the exemplar space can also account for the gradience that has typically been observed in studies of cross-linguistic phonetic influence. By taking into account the distribution of vowels in the production study, exemplar models are also able to account for why the lexical decision results show the asymmetry in error rates between /ɔ/ words and /o/ words. The production data shows that for both groups of speakers (but especially for the Spanish-dominant bilinguals), the production of /o/ in non-cognates is likely to overlap in acoustic space with the production of /ɔ/ in cognates. This pattern in the production data explains the asymmetry in the perception results: when /o/ words are mispronounced with /ɔ/, most of the errors are on non-cognates, because in general, the vowel space for non-cognate /o/ tends to overlap with the vowel space for /ɔ/. Conversely, when /ɔ/ words are mispronounced with /o/, most of the errors are on cognates because the vowel space for /ɔ/ in cognates is much closer to the vowel space for /o/. Exemplar models would assume that past experience with cognate and non-cognate words creates lexically-specific expectations for where these words might fall in the acoustic space, and the results from the lexical decision task reflect that.

## Conclusion

The results of this study indicate that cognate status has an effect on both the phonetic production and processing of the Catalan back mid-vowel contrast by early Spanish-Catalan bilinguals. This cross-linguistic influence was robust for both language dominance groups when producing the experimental stimuli as well as when identifying aurally presented stimuli either as a word or a non-word. Interference at the lexical/phonetic interface has been accounted for in previous studies (Brown and Harper, 2009; Amengual, 2012; Mora and Nadeu, 2012; Brown and Amengual, 2015; Jacobs et al., 2016), but this acoustic interference must be operationalized in a theoretical model that accounts for the observed alterations in the lexical representations of bilingual individuals. This study argues that an exemplar model of lexical representation can be applied to bilingual data to explain cognate effects in which bilinguals do not separate “clouds of memory traces” in each language –they are in fact interconnected– and that the phonetic features of cognate lexical items form a stronger link than non-cognates, thus enhancing cross-language influence. The assumption that the bilingual individual has a single lexicon where lexical elements in different languages are stored together and interconnected has already been proposed in previous bilingual production models (de Bot, 1992). For instance, Hartsuiker et al. (2004) in a study of syntactic priming in bilingual individuals also adopt an integrated view of the bilingual lexicon and make the case for language-specific lexical-syntactic representations, which are then connected to lemma-level representations that are shared between both languages.

While the episodic account provided by exemplar theoretic approaches is reasonable, it is acknowledged that the interpretations provided necessitate further research and support. The extension of this model to include bilingual or multilingual data is intended to open a debate on how the lexical representations and the phonetic abilities of bilinguals interact and how the exemplar model can be extended to include bilingual lexical connections through which cognates facilitate phonetic interference. The study of the mental lexicon either as containing multiple episodes (Goldinger, 1997, 1998; Johnson, 1997a,b; Bybee, 2001; Pierrehumbert, 2001, 2003a,b; Hawkins, 2003) or abstract prototypes (Mehler, 1981; McClelland and Elman, 1986; Pisoni and Luce, 1987; Norris, 1994; Gaskell and Marslen-Wilson, 1997), or a combination of both in a hybrid model holds considerable promise (McQueen et al., 2010). A challenge for future research is to specify which components of the mental lexicon are episodic and which are abstract.

## Author contributions

The author (MA) states that he is solely responsible for the conception or design of the work, and the acquisition, analysis, interpretation of the data, and the drafting of the manuscript.

## Funding

This work was supported by National Science Foundation DDIG # 1226964.

### Conflict of interest statement

The author declares that the research was conducted in the absence of any commercial or financial relationships that could be construed as a potential conflict of interest.
